# Natural Sympathomimetic Drugs: From Pharmacology to Toxicology

**DOI:** 10.3390/biom12121793

**Published:** 2022-11-30

**Authors:** Vera Marisa Costa, Luciana Grazziotin Rossato Grando, Elisa Milandri, Jessica Nardi, Patrícia Teixeira, Přemysl Mladěnka, Fernando Remião, on behalf of The OEMONOM

**Affiliations:** 1 UCIBIO, REQUIMTE, Laboratory of Toxicology, Faculty of Pharmacy, University of Porto, Rua Jorge Viterbo Ferreira, 228, 4050-313 Porto, Portugal; 2 Laboratório Associado i4HB—Instituto para a Saúde e a Bioeconomia, Laboratório de Toxicologia, Departamento de Ciências Biológicas, Faculdade de Farmácia, Universidade do Porto, Rua Jorge Viterbo 228, 4050-313 Porto, Portugal; 3 Post-Graduation Program in Bioexperimentation, University of Passo Fundo (UPF), BR 285, Passo Fundo, São José 99052-900, RS, Brazil; 4 Department of Pharmacology and Toxicology, Faculty of Pharmacy, Charles University, 500 05 Hradec Králové, Czech Republic

**Keywords:** trace amines, adrenaline, cocaine, cathinone, ephedrine

## Abstract

Sympathomimetic agents are a group of chemical compounds that are able to activate the sympathetic nervous system either directly via adrenergic receptors or indirectly by increasing endogenous catecholamine levels or mimicking their intracellular signaling pathways. Compounds from this group, both used therapeutically or abused, comprise endogenous catecholamines (such as adrenaline and noradrenaline), synthetic amines (e.g., isoproterenol and dobutamine), trace amines (e.g., tyramine, tryptamine, histamine and octopamine), illicit drugs (e.g., ephedrine, cathinone, and cocaine), or even caffeine and synephrine. In addition to the effects triggered by stimulation of the sympathetic system, the discovery of trace amine associated receptors (TAARs) in humans brought new insights about their sympathomimetic pharmacology and toxicology. Although synthetic sympathomimetic agents are mostly seen as toxic, natural sympathomimetic agents are considered more complacently in the terms of safety in the vision of the lay public. Here, we aim to discuss the pharmacological and mainly toxicological aspects related to sympathomimetic natural agents, in particular of trace amines, compounds derived from plants like ephedra and khat, and finally cocaine. The main purpose of this review is to give a scientific and updated view of those agents and serve as a reminder on the safety issues of natural sympathomimetic agents most used in the community.

## 1. Introduction

**Figure 1:**
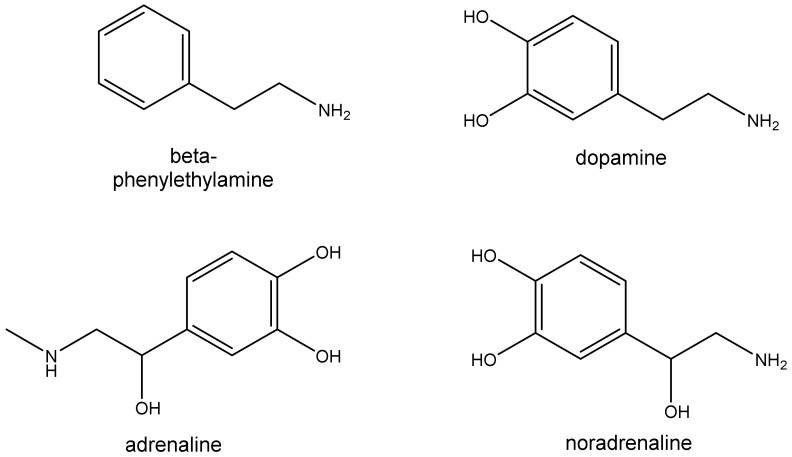
Chemical structure of biogenic catecholamines (dopamine, adrenaline and noradrenaline) and their parent chemical structure (β-phenylethylamine).

In a broad sense, sympathomimetic drugs mimic the action of the endogenous molecules acting on the sympathetic branch of the autonomic nervous system. They are usually classified as catecholamines and non-catecholamines. They increase neurotransmission either directly through adrenoreceptor stimulation or indirectly by increasing and/or prolonging the level of endogenous catecholamines in the proximity of these receptors or by activating intracellular signaling pathways. Catecholamines have β-phenylethylamine as parent chemical structure, which includes hydroxyl groups located at the positions 3 and 4 of the aromatic ring (Figure 1). There are three endogenously occurring catecholamines in humans (dopamine, noradrenaline, and adrenaline) but many amines have been synthetized and used (e.g., isoproterenol and dobutamine) [1,2,3] (Figure 1).

Direct sympathomimetics act as agonists upon one or more adrenoceptors, as their pharmacological activity depends mostly on the subtype, location, and affinity to the adrenergic receptors. Indirect sympathomimetic agents achieve similar outcomes as direct agents on adrenoceptors, but through an increase of endogenous catecholamines in the synaptic cleft. Several mechanisms are known to facilitate those catecholamines increase or surge: displacement of stored endogenous catecholamines from within presynaptic vesicles, inhibition of their reuptake, or inhibition of catecholamine metabolism (e.g., inhibiting their main metabolic enzymes, i.e., monoamine oxidase (MAO) or catechol-o-methyltransferase (COMT)) [1,2,3].

Finally, the presence of catecholamines in circulation or in the neural synapses activates adrenoceptors. Two main classes of adrenoceptors are usually described: α- and β-adrenoceptors. Each group is further subdivided so that five subtypes are presently recognized: two main α-receptor subtypes (α1 and α2) and three β-receptor subtypes (β1, β2 and β3). The α-receptor has six main subtypes (α1A, α1B, α1D, α2A/D, α2B, α2C) but also others have been described [4,5]. Adrenoceptors activate intracellular signal transducers G-proteins and can form homodimers or heterodimers, resulting in different pharmacological and functional properties [4,5], being these areas out of the scope of this revision.

**Figure 2:**
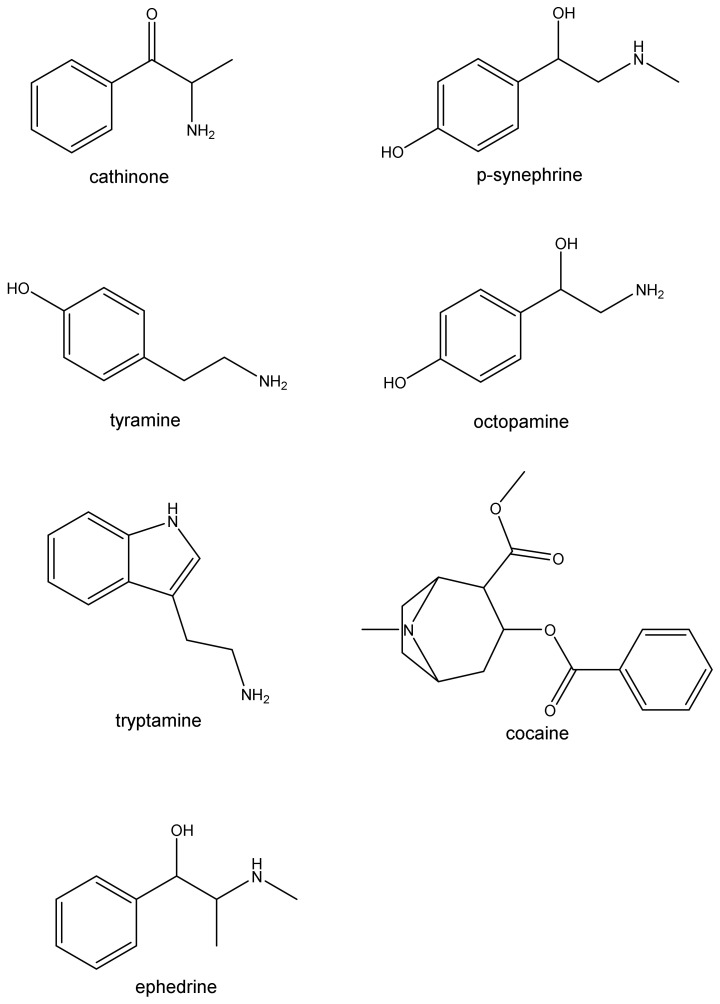
Chemical structures of several natural occurring sympathomimetics.

In the past decades, a newfound interest has emerged on natural compounds and although the lay public has a general belief that naturally occurring agents are safe and innocuous, the current scientific literature shows that they can cause the classic signs and symptoms (‘toxidrome’) often seen after synthetic sympathomimetic drugs. The sympathomimetic syndrome comprises hyperactivity, mydriasis, hypertension, tachycardia, and hyperthermia, among other possibly dangerous effects [6]. Nevertheless, for centuries and in many cultures, naturally occurring sympathomimetic substances have a wide use: in China Ma-huang (ephedrine) from the *Ephedra* plant has been used for 5000 years; in Peru, for 4000 years the Incas chewed the leaves of the coca plant, Erythroxylon coca (cocaine), in their religious ceremonies, while in the region of Andes that cultural habit continues; khat leaves are chewed daily by over 20 million people on the Arabian Peninsula and East Africa, being a deeply rooted cultural tradition of those regions [5], and so on. This ancient use has reinforced the idea of a ‘consequence free’ use of these compounds, which is not the case. In contemporary times, the use of these drugs has found new dosages, routes of administration and even more routine practices, making the risk and adverse effects upon users consistently reported. Thus, this paper aims to shed some light on several natural sympathomimetics, mainly focusing on their pharmacology and toxicology, while reviewing the existent pre-clinical and clinical data to give a critical assessment on their safety. Cathinone, synephrine, cocaine, ephedrine, and trace amines will be mainly addressed for their wide use and/or toxicological potential (Figure 2) in different sections. Although this paper is included in a pedagogic project aiming for undergraduate students mainly in Health Sciences, we aim to give awareness to the lay public against the general notion that natural products are devoid from any toxicity.

## 2. Trace Amines

Trace amines are a chemical group of amines and their isomers, included in the biogenic amines’ family [7]. They are natural compounds with low molecular weight, formed during the natural metabolism of animals, plants, and microorganisms from amino acids precursors [8,9,10]. In general, the trace amine family includes all endogenous amines present at a low nanomolar concentrations in invertebrate and vertebrate systems, like m- and *p*-tyramine, β-phenylethylamine, *p*-octopamine, tryptamine, p-synephrine and N,N-dimethyltryptamine [7]. In invertebrates, they act directly as neurotransmitters [11], while in mammalians their roles are mainly attributed to that of supporting catecholamine actions. Not by chance, trace amines are in different areas of mammalian brain and their distribution is strictly linked to the projection domains of noradrenaline, dopamine and serotonin neurotransmitter pathways [7]. However, ingestion of high amounts of trace amines can lead to toxicity, including indirect sympathomimetic effects resulting in amphetamine-like responses [7,12], which will be discussed further next.

Trace amines are obtained through a direct and selective action of decarboxylases on amino acids via removing the carboxyl group. In β-phenylethylamine, tyramine and tryptamine synthesis, the aromatic L- amino acid decarboxylase (AADC) mediates L-phenylalanine, L-tyrosine, and L-tryptophan decarboxylation, whereas octopamine is formed after hydroxylation of *p*-tyramine by dopamine–β–decarboxylase. In addition, N-methylation represents another pathway by which tryptamine can be metabolized, being that the formation of N,N-dimethyltryptamine is catalyzed by indolethylamine–N–methyltransferase [13].

### 2.1. Sources

Trace amines are naturally present in the human organism, even if their endogenous production does not represent their primary source [14]. In fact, fermented cheese, fermented cabbage, chocolate, raw and smoked meat, raw and fish products, alcoholic beverages, soybeans products are some of the foodstuffs where trace amines have been detected. Their presence and amounts in food varies, which can be affected by intrinsic and extrinsic factors, such as: availability of free amino acids; presence of microorganism with aminogenic capacity, namely decarboxylase-positive microorganisms, and overall conditions that allow microorganisms’ growth [15].

Moreover, food matrixes own different profiles of trace amines, depending on free amino acid and protein amounts in the starting materials [16]. They are usually present in foodstuffs following degradation of proteins and peptides, including proteolysis by microorganisms. Biogenic amines and consequentially trace amines’ production occurs in the presence of microorganisms able to decarboxylate amino acids through substrate-specific enzymes, as stated [8,17]. Decarboxylases are a group of enzymes that belong to the pyridoxal-phosphate-dependent family, where pyridoxal-5′-phosphate represents the coenzyme. Decarboxylase-positive microorganisms are naturally present in food, being often added during the manufacturing processes. Thus, trace amines production depends on the microorganism strain and its’ habitat conditions [18]. Not by chance, differences in the quantitative and qualitative profiles of trace amines have been observed concurrently with microbiological changes, as a result of the variable response of the decarboxylase activity to the environmental factors [19]. According to Gardini et al., temperature, pH and salt concentration represent the most relevant factors affecting biogenic amines quantity in foods, but storage and production processes may also modulate their profile. These conditions act synergically augmenting or reducing the presence of trace amines. Tyramine, and other biogenic amines, are usually predominant during ripening, even if a decrease in their maximum concentration occurs in conformity with variations in the microbiota [20]. Several amines, such as histamine, tyramine and phenylethylamine have been found in different types of ripened cheese, where they are mainly produced and accumulated by lactic acid bacteria or by microorganisms with decarboxylase activity (e.g., *Bacillus*, *Pseudomonas*, *Escherichia*, *Enterobacter*, *Salmonella*, *Shigella*, *Staphylococcus*, *Streptococcus*, *Lactobacillus*, *Enterococcus*, *Lactococcus*, and *Leuconostoc*) [14,19]. Even so, many elements promote or inhibit microbial activity during spontaneous fermentation: the use of food additives, or other synthetic and natural compounds could inhibit the formation of amines [21]. Caraway and onion reduce the amount of the biogenic amine in sauerkraut, depending also on the temperature during its preparation, for instance. The salt concentration is another impacting factor that contributes to a reduction in trace amines in foods, as the activity of decarboxylating microbiota is inhibited by the addition of salt depending on bacteria species present [19]. The following table shows sources and amounts of trace amines in food (Table 1).

### 2.2. Pharmacology

In mammals, the pharmacological proprieties of trace amines are traditionally attributed to support classical biogenic amine activity. Nevertheless, they own specific receptors, Trace Amine—Associated Receptors (TAARs), which are G protein-coupled receptors (GPCR). TAARs own distinctive structural characteristics of the rhodopsin-beta adrenergic receptor superfamily with a chain formed by 7 transmembrane domains [30,31]. At the start of 2001, the TAARs family was discovered through two independent works. Their genes were found in several species, namely humans, rats, mice, and chimpanzees, while interspecies differences in the distribution of these receptors were also found [32,33,34]. After that, their roles have been further investigated, highlighting their importance in both invertebrates and vertebrates. Even if their expression is notable in both invertebrates and vertebrates, only six of them have been reported so far in humans. When facing TAAR2, TAAR5, TAAR6, TAAR8 or TAAR9, only TAAR1 demonstrates a high affinity towards trace amines and will be the only one approached next.

#### 2.2.1. TAAR1 and Its Agonists

TAAR1 receptor is the oldest member known of the TAAR family, and it shows to have interactions especially with β-phenylethylamine, but also with tyramine, tryptamine, octopamine, and the dopamine metabolite, 3-methoxytyramine [13]. In addition, a thyroid hormone derivative, 3-thyronamine (T1AM), and isoproterenol activate TAAR1 too [35]. Moreover, psychotropic agents, such as 3,4-methylenedioxymethamphetamine (MDMA), lysergic acid, amphetamine and methamphetamine are partial agonists to this receptor [12,36,37,38].

Current understanding confirms an interplay between the TAAR1 and the dopamine systems. β-phenylethylamine and tyramine are synthesized in dopaminergic terminals, but are not stored within synaptic vesicles. Thus, they can easily diffuse across membranes. On the other hand, their reuptake into presynaptic terminals has no specific transporter and seems to occur via the organic cation trans-porter 2 (OCT2) [13]. Not by chance, the TAARs family is also expressed in various brain areas, including olfactory epithelium neurons, where it may act as a new class of receptors for different volatile compounds [13]. Moreover, these receptors are present in the crucial brain areas linked to the monoaminergic system, e.g., those implicated in mood regulation, emotion, learning, cognition, attention, and reward [7]. Altered levels of monoamines seem to be involved in various neuropsychiatric disorders, including schizophrenia, depression, attention deficit hyperactivity disorder (ADHD), bipolar disorder and anxiety. Actually, amphetamines cause an increase in the extracellular levels of dopamine in the brain, and the discovery that amphetamine-like compounds acting on TAAR1 can make this receptor as a possible therapeutic target for their side effects [38].

In vitro studies have revealed a probable relationship between TAAR1, monoamine autoreceptors and monoamine transporters [38].

#### 2.2.2. TAAR1 Localization

TAAR1 is primarily located intracellularly both in the Central Nervous System (CNS) and in the periphery, and in neurons and glial cells. Anatomically, considerable overlaps exist between the expression of TAAR1 and dopaminergic and serotonergic brain structures [39]. TAAR1 is also present in the periphery, namely on leukocytes [39], and in the vasculature [13], as well as in the stomach, intestine, kidneys, liver, and pancreas [13]. In addition, TAAR1 appears to regulate hormone release in response to different stimuli and nutrients, assuming thus a role as a novel target for metabolic diseases [40]. Due to its presence on leukocytes, TAAR1 may be a target in inflammatory bowel disease, even if other TAARs seem to be able to activate leukocytes recruitment to the gastrointestinal mucosa and act through other mechanism involved in the outset of that intestinal disease [40,41].

**Figure 3:**
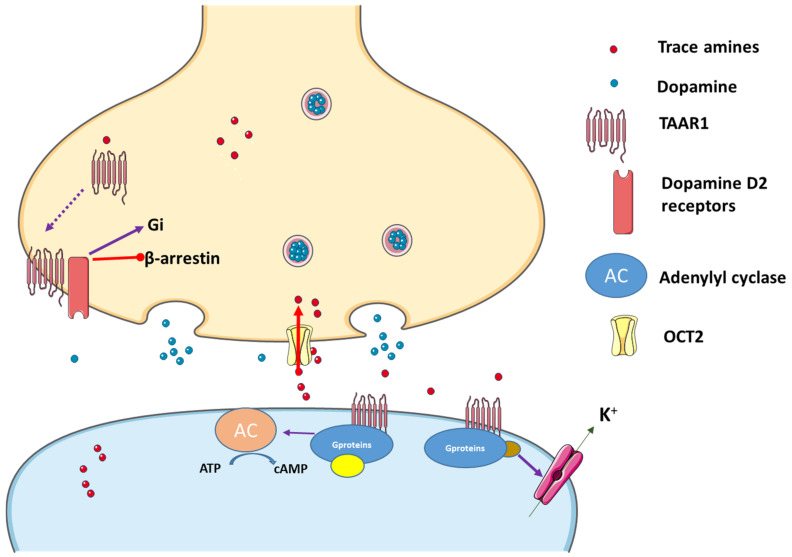
Trace amines activate TAAR1, leading to adenylyl cyclase (AC) activation and downstream activation of protein kinase A and protein kinase C. Interactions with the dopaminergic system can also occur and TAAR1 may translocate to the cell surface after heterodimerization with dopamine receptor 2 (D2), an effect that promotes preferential signaling related to dopamine and through the Gi signal transduction cascade, while blocking the β-arrestin 2 pathway. In addition, K+ channels can be activated in the presence of TAAR agonists. Figure built resourcing to the blocks of Servier Medical Art.

Another important feature of TAAR1 is its predominantly intracellular location. TAAR1 carries out both pre- and postsynaptic effects. Though it is considered a GPCR receptor, and being associated with the cellular membrane, the exact place of TAAR1 in the cell is not yet known, and there is still a need of research in this area [13]. Once ligands bind to the TAAR1 pocket region, different signaling pathways are activated. TAAR1 can couple to Gs and Gq. Hence, upon stimulation, it triggers the accumulation of intracellular adenosine 3′,5′-monophosphate (cAMP) via adenylyl cyclase or phosphoinositol metabolism. Subsequently, protein kinases B and C are activated, promoting extracellular Ca^2+^ influx. Excessive Ca^2+^ influx may lead to oxidative stress acting on the endoplasmatic reticulum and generating mitochondrial dysfunction. TAARs can also operate through β–arrestin 2, a protein associated with their desensitization [42]. Once activated, GPCR regulates various effectors through its subunits (Gα and Gβ) and following their phosphorylation through the action of kinase enzymes, β–arrestin 2 can be recruited. On the other hand, TAAR1 can translocate to the cell surface after heterodimerization with dopamine 2 (D2) receptors, an effect promoted by the Gi signal transduction cascade [13,43], and also involvement of the β–arrestin 2 pathway [43]. In addition, TAAR1 ligands can activate G protein-coupled inwardly rectifying potassium channels (GIRK), which is a family of potassium channels located mostly in the brain, but also in the heart (Figure 3). Once ligands stimulate TAAR1, the heterodimer Gβγ, formed from the GPCR, activates GIRK causing hyperpolarization of the membrane [44]. In summary, when activated, TAAR1 leads to an increase in intracellular cAMP production with protein kinase A and protein kinase C activation and phosphorylation of downstream targets, although other mechanisms can be involved. In fact, it also activates a G protein-independent, β-arrestin 2-dependent pathway involving protein kinase B (AKT)/glycogen synthase kinase-3 (GSK-3). Moreover, it has become evident that TAAR 1 interacts as well with dopamine transporters (DAT) and dopamine D2 receptors [45,46,47]. Nevertheless, phosphorylation of the DAT through TAAR1-stimulated activation can lead to DAT internalization, which leads to reduced dopamine uptake under certain conditions [38] (Figure 3).

#### 2.2.3. Trace Amines as Indirect Sympathomimetic Agents

Before the discovery of the TAARs family, trace amines were mainly considered indirect sympathomimetic agents, causing vasoconstriction, and consequentially increasing blood pressure in vascular beds. It is well-know that a high concentration of trace amines, especially tyramine and β-phenylethylamine, mimics the action of amphetamines. They enter the pre-synaptic terminal neurons causing catecholamine displacement from cytoplasmic pools. Consequently, tyramine and β-phenylethylamine lead to the release of noradrenaline in the synaptic cleft, which causes vasoconstriction by stimulating α-adrenoceptors [12]. In fact, an increase in systolic blood pressure, following a high ingestion of tyramine, is known as the “tyramine pressor response” [48]. Some results suggest that the indirect sympathomimetic effects played by trace amines are not their only mechanisms of action in the vascular system, since they can interact with other receptors [49]. Evidence has been shown that trace amines, particularly tyramine, β-phenylethylamine and octopamine, act directly on α1- adrenoreceptors, generating different responses based on the vessel evaluated. Overlooking the indirect sympathomimetic effect produced by trace amines, the α1-adrenoceptors activation causes intensive vasoconstriction, suggesting a direct interaction between trace amines and the receptor. Tyramine and β-phenylethylamine are also able to promote a contractive response in guinea pig and rat isolated gut preparations, quite the opposite to what occurs in the presence of sympathomimetic amines [50]. Research carried out using a high concentration of an antagonist of TAAR1, called EPPTB (RO-5212773), has shown a reduction in the vasocontractile response in the porcine coronary artery [49]. Additionally, tyramine and β-phenylethylamine exhibit a partial antagonism at β1 and β2 adrenoceptors, while octopamine has shown a partial β1 agonism and a partial orthosteric antagonist in β2 adrenoceptors [35]. The inhibitory effects seem to depend on the doses used and the animal species under evaluation. These data highlight that further research is needed to understand completely the mechanisms of action of trace amines on the TAARs family.

#### 2.2.4. Trace Amines Pharmacokinetics

Regarding their pharmacokinetics, trace amines show a rapid turnover due to their metabolization to biologically inactive products mainly through MAOs. MAOs encompass two different intracellular isoenzymes located in mitochondria, with various substrates and specific organ locations. MAO-A is primary found in the stomach, in the intestine and the gut, in addition to the placenta and sympathetic neurons. MAO-B is predominant in the brain and in the serotoninergic neurons, where β-phenylethylamine represents its selective substrate [13]. Differently from β-phenylethylamine, other compounds do not show a pronounced selectivity towards either MAO-A or MAO-B. For patients with MAOs deficiencies or using MAO inhibitors (MAOI) as drug therapy, an increase of trace amines could contribute to the development of toxicological responses [51], as it will be discussed next.

### 2.3. Toxic Effects of Trace Amines

Though trace amines are found in nanomolar concentrations in the human organism, their toxicology aspects are being debated until now and are even controversial. According to the European Food Safety Authority (EFSA) Panel on Biological Hazard (BIOHAZ), tyramine and histamine are the most common and toxic biogenic amines present in foods, although their risk assessment is still not quantified [51]. Nevertheless, some studies have advocated for their toxicity. Cytotoxicological effects played by tyramine and histamine via a synergic mechanism have been exhibited, when using a human colon adenocarcinoma cell line HT29. Changes in cells homoeostasis, induction of apoptosis and necrosis, have been observed depending on the dose. Tyramine induces necrosis, whereas histamine apoptosis, at concentrations commonly found in food [52]. In addition, below their toxicological concentration per se, histamine potentiated the cytotoxicity of tyramine [53], when it is known that they can both be present in food.

Historically, the first report of trace amines toxicity was associated with hypertension. A woman taking MAOI drugs suffered strong headaches after eating cheese, being these effects thereafter known as “cheese syndrome” [13,54]. Generally, trace amines ingested in the diet are immediately detoxified by MAOs, but in people with dysfunctional MAO or inhibited MAO, they can lead to serious toxicity, mainly in the vascular system [19], as explained. Tyramine, β-phenylethylamine and tryptamine are the trace amines with the highest risk for these acute severe effects. They may generate vasoconstriction responses including headaches and even hypertensive crises, but also vomiting, nausea, pupil dilatation, and breathing difficulties [51]. According to different data reported in BIOHAZ review about biogenic amines, the food—drug interaction is one of the most common causes of toxicity related to trace amines, especially in the case of tyramine. The threshold for tyramine for healthy individuals is established at 600 mg. Above that concentration, tyramine could provoke an increase of almost 30 mmHg in the systolic blood pressure. Conversely, clinical cases regarding intoxications after trace amines are not very frequent in the literature. Most common toxidromes regarding trace amines are related to pharmacokinetic interactions with cheese or other foods in patients who were treated with MAOI drugs. However, IMAOs are not frequently used nowadays in depression therapy. One case report, nonetheless, describes a 34-year-old woman, with a depressive state and in treatment with phenelzine, a non-selective and irreversible MAOI. This woman suffered a myocardial infarct resulting from a MAOI-induced hypertensive crisis, after eating cheese [55]. EFSA also reported data regarding toxicity of trace amines in patients in treatment under Reversible Inhibitors of Monoamine Oxidase (RIMAs), a new generation of monoamine oxidase inhibitors. In these cases, the threshold is higher than for MAOIs, and 150 mg of tyramine is usually well tolerated [51].

Another toxicological effect played by trace amines is migraine. An increase in tyramine and β-phenylethylamine provokes cerebral vasoconstriction and dilatation, causing pain. Usually, the migraine is supported by nausea being seen in susceptible patients after the ingestion of foods rich of tyramine and β-phenylethylamine. Those effects usually begin 1–12 h after ingestion. Their effects are intensified by the consumption of ethanol or coffee, due to the amplification in vascular responses [56]. In addition, ethanol can inhibit MAO too and augments the membrane permeability of the gut, thus increasing trace amines absorption without major metabolization [19]. Tyramine and β-phenylethylamine have also been found in tobacco. Combustion of cigarettes releases compounds that inhibit MAO, contributing to an increase in trace amines and consequentially noradrenaline concentrations by the indirect mechanisms. In smokers, levels of MAO-B and MAO-A are decreased almost by 40%, which can impair metabolism, and potentially lead to interactions but other studies are required [12].

Finally, synephrine is found to have a different toxicological profile regarding trace amines. Due to its particularities, the trace amine synephrine is discussed separately below. In conclusion, though MAOIs have a good profile in safety terms, and technological factors affecting trace amines content in foods are being studied, patients need to limit the intake of foods particularly rich in tyramine to avoid toxicity [19,57], especially if taking drugs affecting their metabolism or having high risk of vasoconstriction complications.

## 3. Synephrine

Synephrine is a phenylethylamine alkaloid found as a trace amine in the human organism [58,59]. There are three different positional isomeric forms of synephrine: ortho o-, meta m- and para p-synephrine (Figure 2), and each isomer can be found in two enantiomeric forms: R-(−) and L-(−). p-Synephrine is recognized as the isomer with natural occurrence. Little is known about o-synephrine. m-Synephrine is also named phenylephrine, being used as an intranasal decongestant [60]. Different isomers present dissimilar activities regarding receptor-binding properties, which naturally affects their pharmacological and toxicological effects [60,61,62].

p-Synephrine is mainly used in herbal products for weight-loss due to its purportedly lipolytic abilities [59,60,61]. This use emerged as a safer alternative when ephedra and related products were banned from dietary products by the US Food and Drug Administration (FDA) in 2004. Ephedrine was linked to serious cardiovascular events [60,63], which will be mentioned in a following section. However, concerns regarding synephrine safety due to its structural and pharmacological similarities with other sympathomimetic amines have emerged with new data [60].

### 3.1. Sources and Amounts Found in Natural Sources

The most common source of p-synephrine is *Citrus aurantium*, a Rutaceae family plant popularly known as bitter orange, Seville orange or zhi shi in Traditional Chinese Medicine. It is mostly found in peels of the unripe fruits, and its concentration decreases as the fruit matures. Moreover, environmental factors such as soil, light, temperature, external stresses, nitrogen and manganese soil levels are believed to influence synephrine amount in fruits [64,65]. In addition, synephrine can also be found in other *Citrus* species such as *C. sinensis*, *C. deliciosa*, *C. limon*, *C. limonia*, *C. unshiu*, *C. bergamia*, *C. tangerine*, and *C. reticulata* and its presence had also been described in *Evodia rutaecarpa* [66,67,68]. In fact, *C. aurantium* is the main natural source of synephrine used in weight-loss dietary supplements [65,66,69,70,71].

Most authors state that only p-synephrine occurs naturally [62,63,68,72,73,74]. Instead, others believe that m-synephrine is also available naturally [75,76], as it has been already detected in *C. aurantium*-based products for weight-loss [71]. The presence of m-synephrine on so called natural products raises concern specially for two reasons: first, the pharmacological and toxicological properties of p- and m-synephrine are substantially different; and second, m-synephrine may have been added to weight-loss products through adulteration [71], in uncontrolled amounts.

A summary of studies evaluating the natural occurrence and found amounts of synephrine is presented on Table 2.

Another possible natural source of synephrine is the honey produced from pollen of orange flowers (a *Citrus* species), the so-called orange honey. Synephrine was detected in concentrations of 79.2 to 432.2 ng/g and is proposed as a botanical marker to attest the authenticity of orange honey [80].

As a trace amine, synephrine was already described in human plasma and platelets of healthy and drug-free males (4.06 ± 3.69 ng/mL and 0.26 ± 0.28 ng/108 platelets, respectively) and females (5.02 ± 5.06 ng/mL and 0.41 ± 0.31 ng/108 platelets, respectively) [81]. p- and m-synephrine were also detected in urine of healthy volunteers who abstained from ingesting citrus fruit and juice for 48 h prior to sample collection (1.8 and 16 ng/mg creatinine, respectively) [82], alleging other natural source(s) and/or endogenous formation.

### 3.2. Pharmacological Effects

Synephrine is structurally similar to adrenaline, noradrenaline (Figure 1 and Figure 2), ephedrine, and amphetamines. These molecules bind to the adrenergic receptors [61,83]. Nevertheless, and despite the structural similarities to sympathomimetic amines, different synephrine isomers present distinct effects. The small structural differences between p- and m-synephrine result in different receptor binding and consequently, diverse physiological and pharmacological effects [62]. Considering the adrenergic receptors, synephrine ligand capacity and properties depend on the substituents of the aromatic ring and of the α and β carbon ligands [59,61,74].

m-Synephrine is considered the most potent synephrine adrenergic agonist at α1 receptors, which explains its’ use as an intranasal decongestant [60]. p-Synephrine exhibits higher selective binding towards β3 adrenergic receptors and has relatively low affinity to α, β1, and β2 receptors [61]. High doses of synephrine directly modify adipocyte metabolism, indicating that these amines may act on human adipocytes and influence fat accumulation if they are ingested in sufficient amounts [84]. Despite the alleged β3 selectivity, p-synephrine has been described as antidepressant in mice, presumably via modulation of noradrenergic neurotransmission [85] and through the stimulation of α1 adrenoceptors [86]. However, to our best knowledge, there are no studies in the recent literature and important interspecies differences exist in adrenergic receptor binding of p-synephrine and other bioamines. The adrenergic receptor binding of p-synephrine in rodents is about 10-fold greater than in humans, thus, the effects observed in rodents cannot be directly extrapolated to humans [84] and that clinical effect is dubious.

The discovery of TAAR genes in humans increased the interest to investigate the role of trace amines as neuromodulators or neurotransmitters [31,87]. In this line, changes in synephrine related pathways have been associated to migraine due to possible abnormalities in neurotransmitters and trace amines and/or abnormal interactions with their receptors [88]. This discovery also provides indirect evidence suggesting that synephrine could induce vascular responses via TAAR receptors [49].

### 3.3. In Vitro and In Vivo Toxicological Effects

There are some noteworthy considerations regarding the toxicological properties of synephrine and synephrine-containing products. The most relevant human exposure is through industrialized products, with *C. aurantium* extract being the most used natural source in dietary supplements. *C. aurantium* presents a variety of bioactive compounds besides synephrine. It has flavonoids, essential oils, limonoids, carotenoids, and other amines as octopamine [62,79]. The presence of these components is likely to influence the properties of *C. aurantium*-based products, making hard a true assessment of the toxicological effects. In addition, these dietary products usually contain stimulants such as caffeine or, sometimes, they are adulterated with synthetic compounds that might trigger severe adverse effects per se or enhance the toxicological properties of the natural product [89,90,91].

p-Synephrine alone has apparently low toxic effects. In calcium-tolerant freshly isolated cardiomyocytes from adult rat incubated with p- or m-synephrine, both positional isomers were internalized. However, only m-synephrine depleted the total and reduced glutathione intracellular levels, suggesting the involvement of oxidative stress in its’ toxicity [92]. Corroborating this hypothesis, the incubation of p-synephrine (2, 20 and 200 µM) for 6 h elicited the overproduction of intracellular reactive oxygen species in hepatic HepG2 cells [93]. Regarding subchronic toxicity, Arbo et al. [94] observed that male albino CF1 mice treated for 28 days with *C. aurantium* extract or p-synephrine presented oxidative stress indicators, but toxicity was considered low. However, when weight-loss products employ high doses of synephrine or synephrine in combination with ingredients such as caffeine, salicin, and ephedrine, the whole mixture can be associated with cardiovascular injury [95] (Table 3).

While p-synephrine is mainly considered to have low toxic potential, because of its low capacity to bind to α1, α2, β1, and β2 adrenergic receptors [61], studies seem to contradict that. For example, mice orally treated with one acute dose of *C. aurantium* extract containing 2.5% p-synephrine (300–5000 mg/kg) or p-synephrine alone (150–2000 mg/kg) presented transitory effects attributed to non-specific adrenergic stimulation [68]. Synephrine also caused vasoconstriction exerted via adrenergic α1 and serotonergic (in particular, 5-HT1D and 5-HT2A) receptors in blood vessels isolated from rat aorta. Unfortunately, the authors did not differentiate which synephrine isomer was employed in this study [67]. Moreover, intracellular glutathione depletion elicited by m-synephrine in calcium-tolerant freshly isolated cardiomyocytes was independent of α1 adrenoreceptor stimulation [92], showing that there are confounding factors related to the role of adrenergic receptor stimulation on synephrine’s toxicity. Binding to TAAR is being pointed as a possible mechanism of action for synephrine beyond adrenergic receptors [49,96].

For systematic purposes, the toxicological effects observed in humans will be described next.

### 3.4. Toxicology and Clinical Cases

The main effects of synephrine-containing products in humans are related to the cardiovascular system and mostly involve products that combine synephrine with other stimulants, as can be observed in Table 3. The Natural Health Products Directorate (NHPD) from Canada adopted a limit of 30 mg/day as the maximum allowable dose for total synephrine content in products, and banned the combination of synephrine and caffeine in dietary supplements [97]. However, in 2011, NHPD revised these recommendations and allowed products providing 40 mg/day or less of p-synephrine plus 320 mg/day or less of caffeine “in order to reduce unnecessary compliance actions on products that do not present as serious a risk to health as had been judged previously” [98]. On the other hand, the French Agency for Food, Environmental and Occupational Health & Safety (ANSES) considers that a dose of 20 mg/day must not be exceeded for food supplements and recommends avoiding combining synephrine with caffeine [99].

**Table 3:** Synephrine and *C. aurantium* related toxicity case reports in humans.

Reference	Route of Administration	Toxidrome	Clinical History
[100]	Oral—Edita’s Skinny Pill 1 capsule daily containing 300 mg of *C. aurantium*, for one year. Other components: carnitine 250 mg, chromium 400 μg, chitosan 250 mg, herbal diuretic complex 200 mg, guarananine 30 mg, green tea 30 mg, calcium 150 mg.	Acute lateral-wall myocardial infarction.	The 55-year-old woman presented dull aching shoulder and chest pain after eating a Chinese food. An arteriogram revealed a lesion in the left main coronary artery (previously unknown), she reported smoking 1 ½ pack per day and high ingestion of caffeine.
[101]	Oral—Stacker 2 Ephedra Free 1 or 2 capsules daily, labelled as having 6 mg of synephrine and 200 mg of caffeine. Treatment underwent for 1 week.	Acute and subacute infarctions in the left thalamus and multiple infarctions in the left cerebellum.	The 38-year-old man presented recent onset of dizziness, difficulty in concentrating, memory loss, and unsteady gait. He had no major risk factors for cardiovascular disease, and had not been taking long-term medications.
[102]	Oral—CortiSlim 1 tablet twice daily. It contains: Leptiplex 125 mg (*C. aurantium* extract with 5% synephrine and Green tea leaf extract with 50% epigallocatechin. Other components: vitamin C 100 mg, calcium 100 mg, chromium 50 mg, insutrol 16.5 mg (Banaba leaf extract and vanadyl sulfate).	Variant angina involving the right coronary artery.	The 57-year-old man presented left-sided chest pressure with radiation to the jaw, shortness of breath, and diaphoresis while at rest. He had history of hypertriglyceridemia and gastroesophageal reflux disease. He had quit smoking 7 years yearlier and reported drinking 3 to 4 alcoholic beverages at night. Long-term medications used: fenofibrate, omeprazole, aspirin, a multivitamin, vitamin B complex, and vitamin E.
[103]	Oral—Lipo 6 twice daily for 3 months. The supplement contains: synephrine, caffeine, and yohimbine (concentrations were not available).	Severe rhabdomyolysis, complicated by acute renal failure and bilateral compartment syndrome.	The 22-year-old, previously healthy, obese (body mass index 31), man, with sickle cell trait presented fatigue, light-headedness, and myalgia that started while running. He was diagnosed with rhabdomyolysis and heat exhaustion, and was discharged after brief hospitalization. Several weeks later, he had another episode of rhabdomyolysis and heat exhaustion, in this case severe. He presented hypotension, tachycardia, tachypnea, and myalgias. Initial evaluation revealed combined lactic and respiratory acidosis with metabolic alkalosis stemming from muscle hypoxia and ischemia with imminent respiratory failure. He developed hypovolemic shock, respiratory failure, acute renal failure, and disseminated intravascular coagulation. The patient had no history of exercise-associated rhabdomyolysis when performing physical activities without dietary supplements.
[104]	Oral—Xenadrine-EFX was used for a few months. Posology was not detailed.	Left middle cerebral artery vasospasm and stroke.	The 36-year-old woman presented weakness in the right upper extremity, difficulty in speaking, right facial droop and severe headache. Exams revealed cerebral infarctions in the left frontal and opercular regions and decreased diameter of the left middle cerebral artery. She was previously healthy, with no history of migraine, tobacco use, thrombophilia, hyperlipidemia, or oral contraceptive use.
[105]	Oral—Hi-Tech Lipodrene 1 tablet twice a day in the first week and then 2 tablets twice a day for two weeks.	Ventricular fibrillation.	The 27-year-old active duty Air Force female was performing the routine physical training when suddenly stopped and lied inactive. She presented thread pulse and agonal breathing, and cardiopulmonary resuscitation was required. The electrocardiogram showed a right bundle branch block and a QTc prolongation. She had a five pack-per-year smoking history and was previously healthy.
[106]	Oral—Nutrex Lipo-6x 1 capsule twice a week for 3 weeks. The supplement contains synephrine, yohimbe, and phenylethylamine. He also reported the use of caffeine energy drinks.	ST-segment-elevation myocardial infarction (STEMI).	A previously healthy 24-year-old man presented acute-onset, “crushing,” mid-sternal chest pain that was accompanied by shortness of breath, ansiety, diaphoresis, and emesis. Emergent coronary angiography revealed extensive, diffuse thrombi in the left anterior descending coronary artery. The patient had no risk factors for coronary artery disease, and denied the use of drugs and alcohol.
[107]	Oral—Jillian Michaels’ Fat Burner (two pills every morning) and Calorie Control (two pills three times a day). She increased the dosages 4 days prior to the episode.	Severe psychosis.	The 52-year-old woman with a history of anxiety, depression, and hypothyroidism, presented a change in mental status, tachycardia, high blood pressure, and unsteady gait. She had abused phenylpropanolamine and over-the-counter diet pills 28 months prior, which led to a previous episode with similar symptoms. She was previously taking buspirone and levothyroxine regularly. She denied drug and alcohol abuse, but her urine drug screen was positive for amphetamines >1000 ng/mL.
[108]	Oral—She had been taking dietary supplements containing caffeine and synephrine during one week. Posology and product were not detailed.	Apical ballooning syndrome.	The 21-year-old female presented disturbed consciousness and seizure. Admission exams evidenced apical ballooning of the left ventricle due to akinesis from the apical to mid ventricular segments, hyperkinesis of the basal segments, with reduced left ventricular ejection fraction. She had no prior relevant medical history.
[109]	Oral—two doses of C4 pre-workout supplement (contains synephrine and caffeine) within one hour of symptom onset. He consumed the supplement and energy drinks chronically.	Ascending aortic dissection.	The 38-year-old male presented syncopal episode and hypotension. Exams revealed ascending aortic dissection extending to the left subclavian artery, tricuspid aortic valve with severe aortic regurgitation, moderate left ventricular dilatation with a mildly depressed ejection fraction, and severe pulmonary hypertension. He had no prior significant medical history.
[110]	Oral—Performix stim-free 1 capsule up to three times a day for one year. He drank 3 scoops of Performix SST for the first time on the afternoon of symptoms.	ST-segment-elevation myocardial infarction (STEMI)	The 22-year-old male presented acute onset of “pressure-like” substernal, non-radiating chest pain associated with shortness of breath and nausea while playing basketball. Angiography revealed acute dissection with thrombosis of the distal left main coronary artery leading into the proximal left anterior descending artery. He had no significant prior medical history and denied excessive caffeine use, smoking, and alcohol intake.

Unfortunately, none of the case reports presented in Table 3 analyzed the concentrations of synephrine and its isomers, and the other components content in the products ingested. Thus, when available, the information was only the one obtained from the labels. As already mentioned, *C. aurantium* products may contain a range of different substances and adulteration has been observed frequently [79,90]. Hence, it is difficult to relate the toxic effects to synephrine alone. Nonetheless, it does not reduce the concern regarding the safety of synephrine-containing products, especially considering that they are ingested mostly for people who already present known cardiovascular risk factors due to obesity or overweight, among other co-morbidities. Besides, people also consume these products before exercising, what can also be considered a risk involving the cardiovascular system. Moreover, caffeine drinks are usually consumed by the whole population and might pose a risk when associated to synephrine-containing products, being that some of these products contain caffeine also.

## 4. Ephedrine

*Ephedra* (*Ephedra sinica*, ma huang) has been used in China for over 5000 years as reported above. Ephedrine, the major active constituent in ephedra, is derived from the aboveground parts of the plant and related species, but it can also be chemically synthesized. Chemically, the structures of p-octopamine (nor-synephrine), p-synephrine and m-synephrine (phenylephrine) are similar to ephedrine (Figure 2), but ephedrine is a phenylpropanolamine derivative that does not contain a para-substituted hydroxyl group [62].

The effects of the *Ephedra* plants are mainly related to the ephedrine-type alkaloids, that were the first active ingredients found that plant and whose amounts determines the pharmacological activities of each species. Those alkaloids are (−)-ephedrine, (+)-pseudoephedrine, (−)-N-methylephedrine, (+)-N-methylpseudoephedrine, (−)-norephedrine and (+)-norpseudoephedrine [111].

### 4.1. Sources and Amounts Found in Natural Sources

Ephedrine is a phenylethylamine alkaloid that can be found in multiples plants of the genus *Ephedra*, one of the largest genera of the Ephedraceae family with approximately 67 species worldwide [112]. This genus is the herbal source of ephedrine and it is often distributed in arid and semiarid regions of Asia, America, southeastern Europe, and Northern Africa [113,114]. Although most *Ephedra* species are used as traditional medicines, only three species, namely, *E. sinica* Stapf., *E. intermedia* Schrenk, and *E. equisetina* Bunge are incorporated in the Chinese Pharmacopeia. Among them, *E. sinica* is the most used [113]. In fact, ephedrine occurs as the main alkaloid present in *E. sinica* Stapf while in *E. intermedia* Schrenk the major alkaloid is (+)-pseudoephedrine. Phenylalkylamine alkaloids, such as (−)-ephedrine, l-methylephedrine, l-norephedrine, and their isomers, commonly exist in *Ephedra* stems. While ma huang—the aerial parts of *E. sinica*—contains higher levels of ephedrine alkaloids and has anti-asthmatic and also stimulant effects, ma huang gem- the root of *E. sinica*—is commonly used as an anti-sudorific [115], corroborating different effects.

Additionally, the *Ephedra* herb has been used in the United States for performance enhancement and weight loss, especially in the mid of the XXth century. Being now a regulated drug, ephedrine use became prevalent in sports to make individuals “super athletes”. Due to misuse of products containing ephedrine, numerous adverse events emerged and, in the late 1960s, the International Olympic Committee and other sports federations banned the use of stimulants such as ephedrine [113].

### 4.2. Pharmacological Effects

Ephedrine is the primary active constituent in *Ephedra*, as stated. Dietary supplements containing ephedra were banned in the United States by the FDA, although they are still being used in traditional Chinese medicine. Ephedrine had been used to treat or prevent hypotension, and has been used for asthma, obesity and narcolepsy. Its illicit use continues over the years [62]. Ephedrine has a molecular structure similar to phenylpropanolamine, methamphetamine and adrenaline. It is a sympathomimetic amine and exhibits multiple mechanisms of action that can consist either of a direct agonist effect on α- and β-adrenergic receptors or particularly of an indirect effect involving the release of catecholamines (noradrenaline and adrenaline) [62].

#### 4.2.1. Activation of α1- and α2-Receptors

In a study using human embryonic kidney (HEK) and Chinese hamster ovary cells, ephedrine alkaloids did not activate α1- and α2-receptors and even antagonized the agonist-mediated effects of phenylephrine and medetomidine on α1- and α2-adrenoceptors, respectively [116]. The blockade of presynaptic α2A- and α2C-adrenoceptors can explain some actions of these alkaloids. In another study with pentobarbitone anaesthetized male Wistar rats, ephedrine led to tachycardia and the pressor response was followed by a small depressor response. On the other hand, animals that underwent sympathectomy had a meaningfully reduced tachycardia after ephedrine. Further, ephedrine contracted the vas deferens, but in vas deferens from sympathectomised rats, ephedrine produced almost no α1A-adrenoceptor mediated tonic response. The authors explained that the pressor response to ephedrine is indirect and may involve actions at α1A-adrenoceptors [117].

#### 4.2.2. Activation of β1-, β2- and β3-Receptors

Li and colleagues demonstrated that ephedrine and pseudoephedrine binds to β2 adrenergic receptors [118]. Furthermore, a study measuring the activity of adenylyl cyclase, associated with cAMP accumulation, compared all 4 stereoisomers of ephedrine. The potency towards activation all 3 β adrenergic receptors was stereoselective, but 1R,2S-ephedrine was the most potent of the four ephedrine isomers on all three human receptors, while 1R,2S- ephedrine was an equipotent β1-/β2-adrenoceptor agonist being the only isomer with a weak partial agonist activity on β3-receptors [119]. On the other hand, ephedrine use has been largely publicized as a natural way to fight obesity and, in fact, in obese subjects, it has led to an increase of the expression of β3 adrenergic receptors in human white adipocytes. These results are consistent with ephedrine’s known ability to facilitate weight loss [120], but the individuals used in that study were given both ephedrine and caffeine. In fact, the thermogenic effect of ephedrine has been reported to be increased by the concurrent administration of caffeine: the combination led to a decrease in body weight when given for 16 weeks [121] and also stearoyl-CoA desaturase genetic expression decreased with the combination. In 3T3-L1 adipocytes exposed to extracts of *Ephedra intermedia* Schrenk, triglyceride deposits were reduced [122]. In that same study, changes in the expression of PPARγ, C/EBPα, and SREBP-1 [122], key players of obesity [123], were observed when the extract was combined with flavonoids and caffeine.

#### 4.2.3. Hypoglycemic Effects

Ephedrine and its derivatives such as (+)-pseudoephedrine, (−)-N-methylephedrine, (+)-N-methylpseudoephedrine, (−)-norephedrine and (+)-norpseudoephedrine are considered hypoglycemic agents because they increase incretins responsible for inducing insulin secretion and for inhibiting the release of glucagon [111]. In an experimental model of type II diabetes induced by streptozotocin, ephedrine showed a hypoglycemic effect together with facilitating the regeneration of the pancreatic islets after their chemical atrophy. These data suggests that ephedrine can help to control insulin secretion and hyperglycemia [124]. Furthermore, Hwa-Won and colleagues performed a study using an extract of *E. pachyclada* Boiss stems, which demonstrated to cause inhibition of α-glucosidase and α-amylase activity, resulting in an antidiabetic activity [125]. In fact, high doses of *Ephedrae aqueous* extract given to rats decreased their body weight [126].

#### 4.2.4. Nasal Vasoconstrictor Effects

The action on adrenoceptors also explains other actions of ephedrine. In the nasal fossae, airflow regulation is dependent of the filling and emptying of the cavernous vein plexuses, which means that the regulation of the mucosal vascular network is fundamental to control the airflow and, consequently, the sensation of obstruction. The regulation of blood circulation in these venous plexuses is achieved through α1 and β adrenergic receptors: α1 receptors are preponderant and the activation of these results in vasoconstriction, while the activation of β receptors results in vasodilation. Hereupon, both ephedrine and pseudoephedrine induce a vasoconstrictor effect, which leads to a relief in nasal congestion. Ephedrine is administered via nasal route and only with a prescription, while pseudoephedrine is available for nasal congestion over the counter [127].

### 4.3. Toxicological Effects

Despite its pharmacological effects, ephedrine misuse has spread around the world, thus leading to the development of adverse effects, including stroke, heart attack, liver damage and drug interactions. Moreover, ephedrine’s neurotoxic effects have also been described. The mechanisms behind ephedrine toxicity are still being studied [111].

Han and colleagues made a study where multiple oral doses varying from 125 to 1000 mg/kg/day of an *Ephedra* herb aqueous extract were daily administered to rats for thirteen weeks. During the study, the death of multiple rats was observed, but only in the group taking the highest dose, suggesting that the *Ephedra* herb aqueous extract is lethal only at high doses. The NOAEL (No observed adverse effect level) was determined to be 125 mg/kg/day [126] of the extract.

#### 4.3.1. Cardiovascular Toxicity

Several deaths from cardiac and cardiovascular events were described in patients taking “Hydroxycut” products, which contain ephedrine [111], being this drug reported as the main responsible for those cardiac effects. Actually, shortly after products were marketed for weight loss under the Hydroxycut label, reports of hepatotoxicity, but also of death due to cardiac and cerebrovascular events in previously healthy patients were described [128]. Furthermore, chronic use of ephedrine containing products has been described to produce hypertension, tachycardia, arrhythmia, acute myocardial infarction, cardiac arrest, or sudden death, hemorrhagic and ischemic strokes [111].

Aiming to study the possible cardiac toxicity of ephedrine, multiple doses between 6.25 mg/kg and 50 mg/kg were orally administered to rats. Various effects were observed, including myocardial ischemia, necrosis and apoptosis, leading either to hemorrhage and sudden death, or ended with inflammation and fibrosis. Based on these pathological findings and the known properties of ephedrine, the cardiac toxicity of this alkaloid is thought to be a result of catecholamine release and binding to α- and β-adrenergic receptors, leading to Ca^2+^ release and changes in electrical and contraction properties of the heart [129]. Other cases reports regarding the cardiac toxicity of ephedrine are enclosed in Table 4.

Other life-threatening toxicity was observed in humans (Table 4). After 60 mg ephedrine, 240 mg caffeine among other compounds, a 36-year-old man was admitted to the emergency room after collapsing during a half-marathon. He had a Glasgow coma score of 12–13, presented with tachycardia and a high heart rate, hypoxic and with a temperature of 39 °C. His pupils were grossly dilated. After initial resuscitation, he was intubated, and transferred to critical care. He subsequently developed rhabdomyolysis, requiring hemofiltration before being discharged from critical care and having further intermittent renal replacement therapy [133], thereafter.

#### 4.3.2. Amphetamine-like Effects and CNS Toxicity

Several amphetamine-like responses are attributed to ephedrine, being that it also acts on dopamine brain levels, as well as interferes with other neurotransmitters [134,135]. Moreover, a study was carried out in order to understand the effects of ephedrine on the vesicular monoamine transporter-2 (VMAT2). After three repeated injections of 25 mg/kg of ephedrine, a reversible decrease in VMAT2 activity was observed. These results corroborated that the impact of ephedrine on VMAT2 function is similar to other catecholamine-releasing agents. This effect was transient, as its impact on intra- and extra-neuronal dopamine levels, which may explain the lack of persistent neurotoxic effects [136].

Other common system studied regarding ephedrine-induced toxicity is the brain, in particular its resemblance or not to amphetamine-like effects and even hyperthermia. At an environmental temperature of 23 °C, single doses (intraperitoneal) of (−)-ephedrine caused marked dose dependent hyperthermia and a dose dependent increase in dopamine levels in the caudate/putamen microdialysate [134]. In that same work, (−)-ephedrine administration was compared to d-amphetamine when given at multiple doses. Multiple doses of either ephedrine or amphetamine caused severe hyperthermia, while the increases in serotonin and glutamate levels produced by these multiple ephedrine and d-amphetamine administrations did not significantly differ. On the other hand, elevation of dopamine levels by d-amphetamine were over 2-fold times what was caused by l-ephedrine in the next hours after administration [134]. Multiple doses of either ephedrine or amphetamine led to low striatal dopamine even 7 days after dosing. However, those dopamine levels were reduced by only 25% or less by ephedrine compared to the 75% reductions produced by amphetamine [134]. Moreover, long-term exposure to ephedrine leads to neurotoxicity and neurobehavioral changes in rhesus monkeys. After 8-weeks exposure to ephedrine, weight loss and induction of behavioral changes, manifested as irritability and behavioral sensitization, were observed. Moreover, in the prefrontal cortex and hippocampus, histological abnormalities included neuronal morphological changes, pyknosis and irregular shapes of neurons. In addition, the levels of corticotropin-releasing factor mRNA and protein were increased in the prefrontal cortex and hippocampus of ephedrine-treated animals [137]. These results suggest that ephedrine causes neurotoxicity although possibly lower than amphetamine-related compounds. Also in rhesus monkeys and after chronic usage of ephedrine, the cognitive control functional loop was weakened. Histopathological changes were also observed, with degeneration and apoptosis of nerves and increase in cAMP response element-binding protein (CREB)-related protein expression in the prefrontal cortex [137].

#### 4.3.3. Hepatotoxicity

In general, weight loss supplements were described as causing hepatocellular and/or cholestatic lesions, accompanied with clinical signs of jaundice, vomiting, abdominal pain in case reports [138]. To understand the mechanism of ephedrine hepatotoxicity, a study in a rat model was carried out. The rats were treated with ephedrine at a daily oral dose of 20 mg/kg or 40 mg/kg for 7 days. After that time, rats treated with ephedrine showed increased hepatic expression of the pro-apoptotic Bax protein and decreased expression of the anti-apoptotic Bcl-2 protein and, consequently, a higher number of apoptotic cells. Ephedrine unbalanced the hepatocellular antioxidant system, as it causes oxidative stress seen as increase in malondialdehyde levels and in the expression of the protein nicotinamide adenine dinucleotide phosphate (NADPH) oxidase 1, as well as decrease in the activities of superoxide dismutase and glutathione peroxidase. Additionally, ephedrine increases inflammatory responses, proved by the higher levels of interleukins (IL) IL-1b, IL-6, and tumor necrosis factor α (TNFα) compared to saline control. In this study, it was also perceived that the (transforming growth factor β) TGF-β/Smad pathway participated in liver injury, evidenced by increased expression of α-smooth muscle actin (α-SMA), TGF-β, and Smad3 in the liver, and decreased expression of Smad7. Moreover, in plasma, aspartate aminotransferase, alanine aminotransferase, alkaline phosphatase, and total bilirubin increased, indicating that ephedrine treatment impaired rats liver function [139].

In conclusion, *Ephedra* related compounds are used in weight supplements and even over the counter drugs with significant toxicity, even in healthy individuals. A false idea of security has been given to those products that are not even subjected to any medical surveillance upon use. Although with a safety net better than drugs as amphetamine, the truth remains that this overly confidence use on these compounds results in several hospitalizations every year.

## 5. Cathinone

### 5.1. Definition and Sources

Khat, scientifically named Catha edulis, is an evergreen scrub or tree found originally in the Horn of Africa and the Arabian Peninsula [1,2]. Nowadays, it is found in several other countries of Eastern and Southern Africa, Southwest Arabian Peninsula and in Afghanistan [140,141,142,143,144]. Khat leaves have been used for centuries in recreational and sociocultural situations, usually in ‘chewing sessions’ because of their psychoactive proprieties. The chemical profile of khat leaves depends on environment, climate conditions, culture and even their harvesting. It contains several sympathomimetic compounds. Of the psychoactive compound(s) that can be found, (2S)-2-amino-1-phenylpropan-1-one, ordinarily known as cathinone, was identified and isolated in 1975, from fresh khat leaves [145]. Nevertheless, cathinone is the major psychoactive compound present in the fresh khat leaves, being cathine and (1R,2S)-2-amino-1-phenylpropan-1-ol [(−)-norephedrine], (Figure 2) the other most abundant active components [146]. Furthermore, cathinone is relatively unstable, as the harvesting and drying procedures can lead to its’ rapid decomposition into cathine and norephedrine [147,148]. On average, fresh khat is reported to contain 36–114 mg of cathinone, 83–120 mg of cathine and 8–47 mg of norephedrine per 100 g of fresh leaves [12]. Actually, cathinone is the most abundant of the alkaloids found in the fresh Catha edulis, being key for the stimulant effects that made khat to be known as a ‘natural amphetamine’ [149,150], as will be mentioned next.

### 5.2. Pharmacology of Cathinone and Its Natural Derivatives

Cathinone, cathine and norephedrine have structurally and pharmacologically comparable proprieties to amphetamine and noradrenaline, acting both on central and peripheral nervous systems [151].

Approximately 100–500 g of khat leaves can be chewed in a single khat session over several hours, where cathinone, the main active alkaloid, is absorbed. After its absorption, it undergoes phase I metabolism, namely a reduction of the β-keto group to an alcohol catalyzed by liver microsomal enzymes, producing cathine and norephedrine [146,152]. Cathinone is rapidly and stereoselectively metabolized and only less than 7% is excreted unchanged in the urine [153]. Indeed, human studies confirmed rapid and extensive metabolism of cathinone into cathine and (−)-norephedrine involving the reduction of β-ketone moiety to the corresponding alcohols by phase I metabolic enzymes. Cathinone is mainly excreted in the urine in the form of its metabolites, with only 7% or less of the absorbed parent compound being found in urine [146,148,154,155]. The average terminal elimination half-time of cathinone ranges between 1.5 to 4.3 h [155,156]. The maximal plasma concentration of cathinone is dependent on the dose ingested and is achieved within 1.5–3.5 h following khat chewing. The psychostimulant activity usually comes after half an hour of chewing and can last for about 3 h [157].

As mentioned, cathinone and cathine are responsible for the major psychoactive and sympathomimetic effects of khat [151]. Cathinone is, by far, the most active constituent of khat being mainly responsible for its stimulating and psychoactive effects. It is a β-keto analog of amphetamine sharing the sympathomimetic effects/cardiovascular effects (e.g., increases in blood pressure, contractile force and heart rate), hyperthermia and mydriasis/ [151,155,158] and amphetamine-like CNS stimulant effects [151,159], by inducing CNS dopamine release [148,160]. In fact, cathinone interacts with the monoamine transporters, both in vitro and in vivo [157,159,161,162,163,164,165,166,167,168,169]. Cathinone promotes behavioral effects through transporter-mediated release of the monoamine neurotransmitters dopamine, noradrenaline and/or serotonin [170]. Cathinone is a noradrenaline uptake transporter inhibitor and exhibits selective inhibition at DAT when compared to serotonin uptake transporter (SERT) [171]. In rat brain synaptosomes, stereoselectivity was the key factor for the neurochemical proprieties of cathinone. Cathinone enantiomers differed in potency [being S(−) higher than R(+)], but both enantiomers were >50-fold selective at promoting monoamine release through DAT vs. SERT [170]. The high DAT/SERT ratio showed by these substances points towards their high abuse potential [157]. In rat cerebral cortex membranes, the potency for inhibiting the uptake of noradrenaline by cathinone was very similar to that observed for cocaine [171].

Cathinone can inhibit MAO, with preference towards MAO-B, which subsequently results in dopamine synaptic accumulation [157,172,173]. Enhanced activation of dopaminergic pathways by cathinone in specific areas of the brain is linked to the euphoric effect of khat [157]. Khat chewers describe a feeling of well-being, which is mainly attributed to cathinone, namely increased energy and excitement, an euphoria sensation, increased alertness, enhancement in self-esteem, increased ability to concentrate, an increase in libido, enhanced imaginative ability, improvement in the ability to communicate, capacity to associate ideas, and subjective improvement in work performance [149,157].

Apart from CNS effects, the accumulation of noradrenaline in peripheral sympathetic synapses after cathinone causes effects on other innerved organs [171]. Moreover, cathinone modulates airway tone. Cathinone inhibits electric field stimulation-induced acetylcholine release and the contractions of smooth muscle, possibly via the activation of pre-junctional α2 adrenergic and 5-HT7 receptors [174].

### 5.3. Toxicology of Cathinone and Its Natural Derivatives

Several in vitro and pre-clinical studies were performed to unleash the toxicological effects of cathinone. Khat chewers can experience some negative experiences, such as over-talkativeness, over-activity, insomnia, irritability, anxiety, agitation, aggression, and short-lived schizophreniform, psychotic illness, mania and, more rarely, depression [149,175]. Moreover, chronic use of khat increases the incidence of severe cardiac, neurological, psychological and gastrointestinal complications [157]. Khat consumption is suggestive of that induced by stimulants of the amphetamine type, namely the psychotic behavior observed. Cathinone produces dose-dependent hyperthermia, with increased locomotor activity and rearing behavior [176]. Cathinone use causes extreme restlessness and tremor, hypermotility, stereotyped oral activities, such as compulsive chewing, gnawing and licking among other behavioral features [151]. The relative reinforcing effects of cathinone was studied in rhesus monkeys and it was revealed that the reinforcing efficacy of d,l-cathinone was equivalent to that of cocaine [177]. Moreover, cathinone induces region-specific c-fos expression in the brain [176], which has been linked to dopamine pathways dysfunction [178].

Cathinone causes coronary vasoconstriction, negative inotropy and negative chronotropy in isolated hearts, while its major metabolite norephedrine provokes coronary vasoconstriction comparable with that of cathinone. Conversely, norephedrine has no observable effect on cardiac force or rate [179]. Nonetheless, in the anaesthetized rat, cathinone produced marked tachycardia, mostly abolished by a β-antagonist [180] and not by noradrenaline reuptake transporter inhibition [180], showing that cardiac β-adrenoceptor mediated actions of cathinone are probably largely indirect. Indeed, potency of cathinone to inhibit uptake of noradrenaline was similar to cocaine in rat left ventricular slices [171]. It is likely that cathinone possibly acts on competitive blockade of the noradrenaline transporter rather than simply displacing noradrenaline from vesicles [181]. Since khat consumption is linked to an increased incidence of myocardial infarction, these results may have implications in terms of cardiac morbidity. Khat abuse has direct effects on the cardiovascular system most likely due to the indirect sympathomimetic activity of cathinone, causing increased heart rate and blood pressure in humans, which persists for 3 to 4 h after the onset of khat chewing [155,156]. Khat users, by typically chewing khat and smoking it, have a significantly elevated mean diastolic blood pressure [182]. In addition, there is evidence of enhanced risk of acute myocardial infarction and cardiac arrhythmias among khat users, especially in persons who are susceptible to them [183,184].

Other less described effects of cathinone are worth mentioning. Hypermotility caused by cathinone in hypophysectomized rats was analogous to that reported for (+)-amphetamine in such animals [185]. Moreover, cathinone causes hormonal alterations, namely on cortisol and prolactin levels, possibly via changes in hypothalamo-hypophyseo-adrenocortical and gonadal axes integrity [186].

Other recent works even in our group reviewed both Khat and synthetic cathinones [157,187] are worth looking for further and more detailed information.

## 6. Cocaine

Cocaine is chemically known as benzoylmethyl ecgonine (Figure 2), being a natural alkaloid derived from the coca plant. It is one of the most abused illicit drugs presently. Cocaine is used by inhalation, nasal insufflation, and intravenous injection, but coca leaves have been chewed for thousands of years in South America [188]. Its toxicity has been well reported and its’ sympathothicomimetic actions make it a key drug to be mentioned herein.

### 6.1. Sources

The leaves of the coca plant Erythroxylon coca are originated from the high lands of the Andes and have been used as stimulants in South America for over 4000 years. Cocaine is the psychoactive alkaloid of the coca plant. For Incas, it was a medicinal and sacred plant, being part of religious rituals, prophecies, wedding ceremonies, funerals and ritual initiations of young nobles. Although cocaine isolation occurred only in the mid-1800s, it had been considered safe for a long time and was even used in toothache drops, nausea pills, energy tonics, and, even on the original “Coca-Cola” recipe until 1903 [189]. Presently, its illicit use in western countries largely overcomes its’ sporadic use as anesthetic eye drop [189], as discovered by Koller.

### 6.2. Pharmacology of Cocaine and Its Metabolites

The duration of cocaine effects varies with the way of administration or usage, as well as the form of cocaine (cocaine hydrochloride or crack cocaine, the cocaine base) used, and the concomitant use of other drugs. The cocaine’s onset of effects is most rapid after inhaled cocaine (3–5 s) followed by intravenous injection (10–60 s), and intranasal use (within 5 min) due to topical vasoconstriction [188]. Cocaine is rapidly metabolized by plasma and liver cholinesterases to the major metabolites, benzoylecgonine and ecgonine methyl ester highly excreted in the urine [188]. For further detail on the pharmacokinetics of cocaine see a recent review [190].

**Figure 4:**
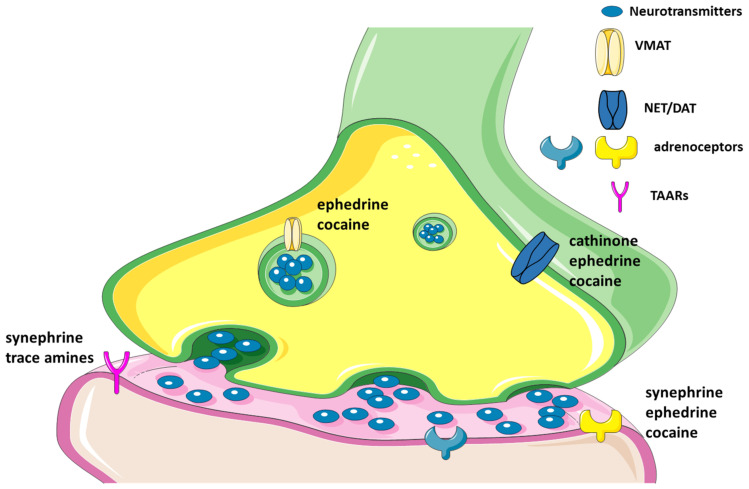
Interactions on the synaptic cleft of several sympathomimetics. Figure built resourcing to the blocks of Servier Medical Art.

Cocaine blocks the reuptake of catecholamines (dopamine, noradrenaline) and serotonin at similar concentrations [191]. Increased serotonergic activity may be involved in the addiction and reward effects of cocaine [192]. Excess dopamine activity, however, is believed to be the principal culprit for central symptoms, either the “good” (desired) and or the “bad” (toxic) effects, which will be later addressed. Cocaine use leads to euphoria, increased self-confidence and sexual drive, and alertness [190,192]. Although serotoninergic pathways are involved, a work in laboratory animals showed that cocaine-induced dopamine overspill in the nucleus accumbens seems to be related to the addictive proprieties of cocaine, being that α1- and β-adrenergic receptors are probably involved in the loss-of-interest to day-to-day important activities when cocaine addiction occurs [193] (Figure 4). Dopaminergic pathways are mostly involved in the central effects of this drug. Cocaine alters dopamine pathways by (1) inhibiting the plasmalemmal DAT as the principal dopaminergic site of action for cocaine; and (2) abruptly and rapidly alters vesicular monoamine transport through VMAT-2 [194]. These effects increase the amount of dopamine available to act at receptors in the synapse [194]. Furthermore, TAAR1 activation changes the response towards cocaine as it reduces cocaine-induced dopamine overflow in the nucleus accumbens at least in vitro [195]. Moreover, TAAR1 activation prevents the effects of cocaine on dopamine transmission, specifically on its uptake [196].

### 6.3. Toxicity of Cocaine

Although cocaine use aims the CNS, as give reproduce the sense of euphoria, wellbeing, improved concentration and self-confidence, it leads to a wide variety of other physical, psychological, and behavioral effects, pending on the user’s profile, route of administration and dose [190]. In fact, CNS symptoms of cocaine include euphoria, increased self-confidence, and alertness at lower doses and aggressiveness, disorientation, and hallucinations at higher doses [192], being attributed to excessive dopaminergic activity [188].

The major acute toxicities related to cocaine abuse result from the sympathomimetic effects. Nevertheless, local anesthetic effects associated with blockade of voltage-gated sodium channels in the neuronal membrane and resulting in inhibition of neural conduction may also be important factors [188]. In detail, tachycardia, hypertension, hyperthermia, diaphoresis, tremors, seizures, mydriasis, headaches, abdominal pain, muscle hyperactivity, hemorrhagic stroke, dysrhythmias, acute myocardial infarction and even multiorgan failure, are often described after acute use [190,197,198]. Nevertheless, chronic use has been linked to neurodegeneration, and premature brain aging, depression, as well as increased risk of chronic cardiovascular diseases [190]. Moreover, the risk of stroke is twice as high in cocaine abusers, compared to age-matched naïve individuals, possibly because it causes cerebral vasospasm and increase in circulating endothelin-1. It has been reported also that cocaine use causes cervicocephalic or intracranial arterial dissection, which are other possible factors for cocaine-induced stroke [198].

The vasoconstrictive effect of cocaine is primarily due to the stimulation of α1-adrenergic receptors [199], although increased endothelin-1 and decreased nitric oxide blood concentrations may also contribute to cocaine’s vasoconstrictive properties [200,201,202]. Cocaine also stimulates the human cardiovascular system via a central mechanism of action [203] involving adrenoceptors.

Although cocaine is rapidly and intensively taken up, as well as cleared from the heart, advocating for a short overall stay of cocaine in the heart, prolonged inhibition of the noradrenaline transporter has been shown, thus demonstrating that the effects of cocaine on cardiac neurotransmitter activity can persist long after the drug has been cleared [204]. This suggests an important role of cocaine metabolites on its pharmacological (and toxicological) mechanisms [190]. For instance, benzoylecgonine showed negligible retention in heart in that study [204]. But, the major metabolites of cocaine, benzoylecgonine and ecgonine methyl ester, may persist in the body for more than 24 h and hence contribute to delayed or recurrent coronary or cerebral vasoconstriction [188] or other processes.

Moreover, prothrombotic properties can contribute to observed cardiovascular toxicity [188]. In detail, cocaine exposure causes platelet activation with their alpha granule release, and platelet containing microaggregate formation [205]. The thrombogenic effects of cocaine are related as well to a rise in plasminogen activator inhibitor activity and increased platelet count. Elevated C-reactive protein, von Willebrand factor, and fibrinogen concentrations can further promote cocaine-induced thrombosis [188,205,206,207]. Interestingly, benzoylecgonine and cocaethylene are even more potent than cocaine to increase endothelial von Willebrand factor release [207]. That can even increase the thrombogenic effects since they have a longer half-life in the blood than cocaine.

In summary, cocaine has a broad range of toxic effects, possibly affecting all body systems. Patients with acute cocaine toxicity symptoms may require urgent treatment for life threatening tachycardia, dysrhythmia, severe hypertension, coronary vasospasm, cocaine-induced rhabdomyolysis, and fetal/maternal morbidity and mortality. Central symptoms like seizure, hyperthermia, euphoria, agitation, delirium, sometimes hallucinations and psychosis can also arise after cocaine use [188,189]. Although dopaminergic and serotoninergic effects are considered the major culprits for cocaine’s effects, the impact on excitatory amino acids or other neurotransmitters (glutamatergic system, muscarinic, and sigma receptors) are also believed to contribute to CNS toxicity [192].

A recent work has been published and is worth looking for further and more detailed information regarding the detection and pharmacology, toxicology and several pharmacokinetic aspects of cocaine [190].

## 7. Caffeine and Other Anti-Thermogenic Drugs

Catecholamines are the only hormones with pronounced lipolytic action in man. Changes on adrenoceptors, signal transduction or even genetic changes have implicated on obesity [208]. Several drugs are used in combination to increase weight loss. In a summary, some natural drugs will be mentioned that relate with sympathomimetic agents. Caffeine is considered a thermogenic agent used in body weight reduction in combination with other drugs already mentioned in this review, such as ephedrine or synephrine. Caffeine-related thermogenesis actions are usually related to the inhibition the phosphodiesterase-induced degradation of cAMP, with minor contribution of antagonism at adenosine receptors [209]. This molecule combined with catecholamine releasing agents or acting on adrenoreceptors like ephedrine can increase weight loss. Nevertheless, caffeine has cardiovascular effects that can increase the risks, mainly cardiovascular of ephedrine, with potential danger to patients. In a study, nine healthy, young, non-coffee drinkers maintained in sodium balance throughout the study period were given caffeine (250 mg) or placebo. Caffeine increased plasma renin, noradrenaline, and adrenaline. Caffeine increased mean blood pressure, accompanied by a slight fall and then a rise in heart rate [210].

Green tea has been widely consumed in China and Japan for many centuries, being regarded as safe. Nowadays, green tea has also been used as a thermogenic agent, containing several polyphenolic components, and also caffeine. Nonetheless, energy expenditure and fat oxidation in humans given green tea is greater than an equivalent amount of caffeine [211]. The other components possibly responsible for these observed effects are flavonoids, like epigallocatechin gallate and other catechins. The catechins in green tea may stimulate thermogenesis and fat oxidation through inhibition of COMT and MAO [212,213,214], enzymes involved on the degradation of catecholamines [4].

Consumption of spicy foods containing capsaicin or non-pungent peppers rich in capsiate, augment energy expenditure and enhance fat oxidation, especially at high doses [215]. It stimulates the sympathoadrenal system that mediates the thermogenic and anorexigenic effects of capsaicinoids. Capsaicinoids have been found to accentuate the impact of caloric restriction on body weight loss. Some studies have also shown that capsinoids, the non-pungent analogs of capsaicinoids, increase energy expenditure. Capsaicin supplementation attenuates or even prevents the increase in hunger and decrease in fullness as well as the decrease in energy expenditure and fat oxidation, which normally result from energy restriction [216]. Capsaicin and capsaicin analogs increase adrenal catecholamine secretion [217,218], which in turn promote thermogenesis through their actions on adrenergic receptors. Also actions on transient receptor potential vanilloid subfamily 1 (TRPV1) may mediate thermogenic effects [215,216] of these compounds. Moreover, the thermogenic effect of capsaicin has been reported to mediated, at least in part, by some capsaicin-sensitive structure in rostral ventrolateral medulla [219].

## 8. Conclusions

At the present times, a common and dangerous notion is gaining strength, being that natural products are safer than others. Special caution should be paid when addressing health issues with compounds interfering, mimicking or exaggerating actions of endogenous molecules. Furthermore, one cannot forget or overlook that we live in a complex world where we make contact with common everyday life chemicals (pesticides, food additives, life-style products components), even at levels well below what is considered hazardous [220]. However, those real scenario mixtures together with consumption of drugs like the sympathomimetics that comprise acknowledged toxicity, can considerably augment the health risks. In the case of synephrine for instances, the adulteration of natural products shows the toxic potential of the combination of several compounds [60]. In fact, we have mentioned several potential ‘toxic combinations’ when we referred to trace amines for instances. In fact, the combination of ephedrine and/or caffeine and synephrine has been widely described as toxic, especially toward the cardiovascular system. To add, the cardiovascular toxicity of natural (and non-natural) drugs needs to be looked with closer attention. First because there is a general lack of legislation and clear identification to what are cardiotoxic compounds in daily life [221] and, second, because all the sympathomimetics (even the ones coming from natural sources as the ones mentioned herein) have a general predisposition to elicit cardiovascular cardiotoxicity.

In addition, when any drug induces desirable, euphoric and reinforcing effects, it may, to some extent, result in abuse and addictive potential. Regarding the sympathomimetics mentioned in this work, while cocaine addictive potential is recognized for several years [190], a new vision on cathinones has emerged with recent studies that recognize their abuse and addictive potential [177,222]. Actually, the addictive potential of some of these compounds adds another danger to the equation as chronic use will be likely and the set of toxic effects will increase.

Finally, we have shown in this review that regardless of the nature of the compound, its action can lead to severe and life-threatening complications. That awareness needs to be transmitted to the general society by overcoming barriers of communication and also some fake notions that are hard to fight back. The CNS and the cardiovascular system, possibly for their intricate nature and richness on catecholamines and their receptors, are the most attained and affected by the sympathomimetic drugs (Figure 4). The notion of ‘natural is safe’ needs to be reassessed and populations need to be aware of the social and economic impact of the misuse of these sympathomimetics.

## Figures and Tables

**Table 1:** Major trace amines tyramine (TYR), β-phenylethylamine (PEA), and tryptamine (TRYP) found in food.

Sample	Trace Amines (mg/kg)			Reference
	TYR	PEA	TRYP	
Soppresata	8.12–511.44	10.56–19.90	ND	[22]
Sausages	10.33–338.85	ND	ND	[22]
Salami	77.14	3.2	1.63	[23]
Hamburger	1.5–27.0	ND	9.3–13.5	[24]
Chocolate	3.11	2.67	1.43	[23]
Coffee	1.26–16.41	/	/	[9,25]
Fermented cabbage	60.66	0.73	0.18	[23]
Azeitão cheese 30 days of ripening	122	/	≅ 65	[20]
Parmigiano cheese	3.75	0.2	0.07	[23]
Yogurt	ND	0.001	ND	[23]
Leerdamer	ND	0.005	0.04	[23]
Red wine	1.93	0.61	0.03	[23]
Red wine Abruzzo (Chieti, Italy)	11.94 ± 7.79 mg L^−1^, mean ± s.d	ND	/	[26]
Rosé wine Abruzzo (Chieti, Italy)	0.27 ± 0.23 mg L^−1^, mean ± s.d.	0.16	/	[26]
White wine Abruzzo (Chieti, Italy)	ND	ND	/	[26]
Fiore Sardo Sheep Cheese (Sardinia, Italy)	350 ± 300 mg/kg, mean ± s.d	/	/	[27]
Lupin Luteus Spontaneously fermented	32.6	128.8	/	[28]
Lupin Albus Spontaneously fermented	69.1	144.1	/	[28]
Soybean Rudoji Spontaneously fermented	30.1 ± 3.1	230.1 ± 8.6	/	[28]
Soybean Progress Spontaneously fermented	27.8 ± 3.2	234.5 ± 12.3	/	[28]
Feta cheese	0–246	0.77–4.94	2.18–6.24	[29]

ND: not detected; /: no information.

**Table 2:** Content of synephrine found in different *Citrus* samples.

Reference	Sample	Synephrine Content
[75]	Seville orange (*C. aurantium*) juice	56.9 ± 0.52 μg/mL
[66]	Dried fruits of *Citrus* species	0.11–2.0 mg/g dry weight
[68]	Unripe fruits and leaves of *Citrus* species	Fruits: 0.037–0.197% Leaves: 0.006–0.087%
[77]	*C. aurantium* fruits and peels	Fruits: 0.99 ± 0.05 mg/g Peels: 1.14 ± 0.02 mg/g
[64]	Juices of peeled *Citrus unshiu* fruits	73.3–158.1 mg/L
[78]	*C. aurantium* fruit extracts and fruit parts	Whole fruit extract: 600 µg/g
		Exocarp: 1100 µg/g
		Mesocarp: 580 µg/g
		Endocarp + juice: 94 µg/g
[79]	*Citrus reticulata* ‘Chachi’ pericarp harvested at different stages	Early harvest: 1.90 ± 0.26 g/kg
		Middle harvest: 1.77 ± 0.27 g/kg
		Late harvest: 1.50 ± 0.20 g/kg
[65]	*C. aurantium* fruits, leaves, stems, and roots in different harvest periods (June–September)	Fruits
		June: 3.36 ± 0.64 g/kg
		September: 0.41 ± 0.04 g/kg
		Leaves
		June: 1.86 ± 0.31 g/kg
		September: 1.08 ± 0.15 g/kg
		Stems
		June: 0.10 ± 0.00 g/kg
		September: 0.49 ± 0.23 g/kg
		Roots
		June: 0.03 ± 0.01 g/kg
		September: ≤ 0.01 g/kg

**Table 4:** Ephedrine related cardiovascular toxicity case reports in humans.

Reference	Route of Administration	Concentrations	Toxidrome	Clinical History
[130]	Oral—cough mixture containing ephedrine.	Consumption of more than 400 mg of ephedrine a day. Ephedrine plasma concentration was not evaluated.	Cardiomiopathy	The 35-year-old man presented with general fatigue and shortness of breath. An electrocardiogram showed sinus tachycardia of 110 beats per min and left ventricular hypertrophy. Chest radiographs showed generalised cardiomegaly. Depressed left ventricular function with a corrected left ventricular ejection time of 65%. The man had had exercise-induced and hyperventilation asthma since the age of 14. In 1972 and 1973 he was treated for cardiac failure.
[131]	Oral—Tablets containing 10 mg ephedrine and 100 mg caffeine. Morning of the presentation day: 20 mg ephedrine 200 mg caffeine Afternoon of the presentation day: 30 mg ephedrine 300 mg caffeine The day before presentation (2 pills morning and afternoon): 40 mg ephedrine 400 mg caffeine	Ephedrine plasma concentration of 150 ng/mL.	Cardiac Ischemia	The 22-year old woman presented with palpitations, nausea, tremulousness, abdominal pain, and vomiting. The initial electrocardiogram revealed a ST segment depression of 1 mm in leads V3 and V4, along with inverted T waves in leads V1–V4.
[132]	Oral—Two tablets (one in the morning and one at lunch) making up a daily ingestion of 24 mg ephedrine and 80 mg caffeine for one month.	Ephedrine plasma concentration was not evaluated.	Myocardial infarction	The 45-year-old woman presented complaining of a 2-h substernal chest pressure with the pain radiating down into both forearms. An electrocardiogram showed a T-wave inversion and nonspecific ST changes suggestive of possible ischemia. The patient smoked 30 packs of cigarettes/year. Her family history was positive for premature coronary artery disease (her father underwent coronary artery bypass grafting at age of 63). Cardiac enzyme measurements revealed an elevated troponin level of 9.6 ng/mL. Cardiac catheterization was performed and no atherosclerotic disease was found in the coronary vessels, thus leading to the conclusion that vasospasm induced by ephedrine alkaloids was the cause of the patient’s acute myocardial infarction.
[133]	Oral— ephedrine 30 mg, as well as aspirin 30 mg, caffeine 120 mg and narnegin 80 mg.	Ephedrine plasma concentration was not evaluated	Collapse	Male with collapse after running a half-marathon. On hospital arrival, a Glasgow coma score fluctuating between 12 and 13, hypoxic, and a temperature of 39 °C. Tachycardia and bilateral mydriasis. After being resuscitated, became hypoglycaemic and suffered two seizures. After intubation, he was admitted to the critical care unit.

## Data Availability

Not applicable.

## Abbreviations

AADC	L- amino acid decarboxylase
CNS	Central nervous system
DAT	dopamine active transporter
GPCR	G protein-coupled receptors
EFSA	European Food Safety Authority
FDA	The U.S. Food and Drug Administration
MAO	monoamine oxidase
MAOI	monoamine oxidases inhibitor
TAARs	trace amine—associated receptors
VMAT 2	vesicular monoamine transporter 2
